# 1-[(3-Benz­yloxy-2-nitro­phen­oxy)meth­yl]benzene

**DOI:** 10.1107/S1600536812029194

**Published:** 2012-07-04

**Authors:** Hoong-Kun Fun, Suhana Arshad, S. R. Ubaradka, Prakash Shetty, Arun M. Isloor

**Affiliations:** a X-ray Crystallography Unit, School of Physics, Universiti Sains Malaysia, 11800 USM, Penang, Malaysia; b Chemistry Department, Manipal Institute of Technology, Manipal, India; c Department of Printing, Manipal Institute of Technology, Manipal 576 104, India; d Department of Chemistry, National Institute of Technology-Karnataka, Surathkal, Mangalore 575 025, India

## Abstract

The asymmetric unit of the title compound, C_20_H_17_NO_4_, consists of two crystallographically independent mol­ecules. In one of the mol­ecules, the central benzene ring forms dihedral angles of 2.26 (6) and 58.68 (6)° with the terminal benzene rings and the dihedral angle between the terminal benzene rings is 56.45 (6)°. The corresponding values for the other mol­ecule are 35.17 (6), 70.97 (6) and 69.62 (6)°, respectively. In the crystal, an inversion dimer linked by a pair of C—H⋯O hydrogen bonds occurs for one of the unique mol­ecules. C—H⋯π and π–π [centroid–centroid distances = 3.7113 (8) and 3.7216 (7) Å] inter­actions link the components into a three-dimensional network.

## Related literature

For background to 1-((3-(benz­yloxy)-2-nitro­phen­oxy)meth­yl)benzene derivatives, see: Altmann *et al.* (2004 ▶); Ohkubo *et al.* (1997 ▶). For related structures, see: Naveenkumar *et al.* (2009 ▶); Fun *et al.* (2011 ▶); Ren & Wang (2012 ▶). For the stability of the temperature controller used in the data collection, see: Cosier & Glazer (1986 ▶).
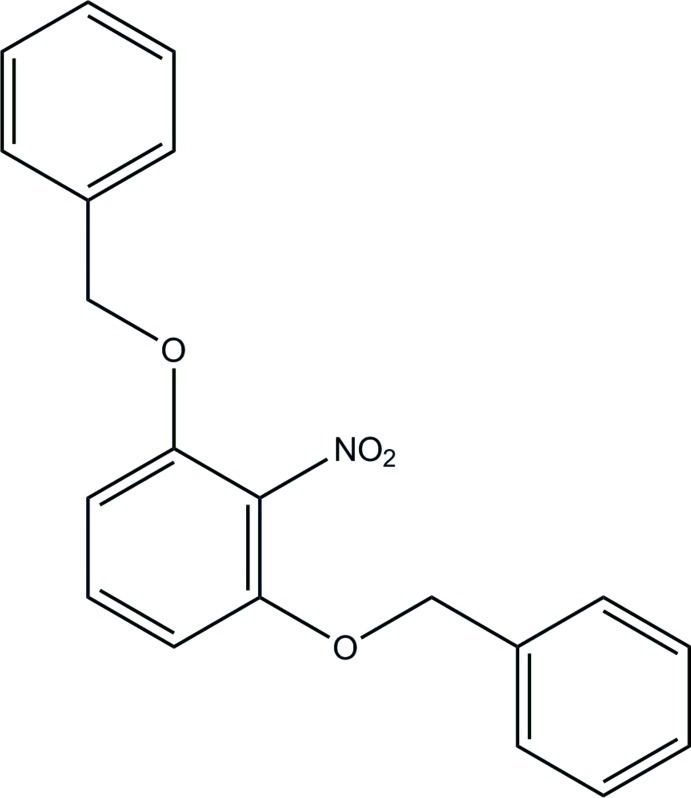


## Experimental

### 

#### Crystal data

C_20_H_17_NO_4_
*M* *_r_* = 335.35
Triclinic, 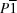

*a* = 7.6150 (4) Å
*b* = 14.6248 (7) Å
*c* = 15.2915 (8) Å
α = 94.706 (1)°
β = 101.627 (1)°
γ = 90.572 (1)°
*V* = 1661.80 (15) Å^3^
*Z* = 4
Mo *K*α radiation
μ = 0.09 mm^−1^
*T* = 100 K
0.26 × 0.19 × 0.09 mm

#### Data collection

Bruker SMART APEXII DUO CCD diffractometer
Absorption correction: multi-scan (*SADABS*; Bruker, 2009 ▶) *T*_min_ = 0.976, *T*_max_ = 0.992
35546 measured reflections
9716 independent reflections
7508 reflections with *I* > 2σ(*I*)
*R*_int_ = 0.032

#### Refinement

*R*[*F* ^2^ > 2σ(*F* ^2^)] = 0.042
*wR*(*F* ^2^) = 0.117
*S* = 1.03
9716 reflections
451 parameters
H-atom parameters constrained
Δρ_max_ = 0.38 e Å^−3^
Δρ_min_ = −0.29 e Å^−3^

### 

Data collection: *APEX2* (Bruker, 2009 ▶); cell refinement: *SAINT* (Bruker, 2009 ▶); data reduction: *SAINT*; program(s) used to solve structure: *SHELXTL* (Sheldrick, 2008 ▶); program(s) used to refine structure: *SHELXTL*; molecular graphics: *SHELXTL*; software used to prepare material for publication: *SHELXTL* and *PLATON* (Spek, 2009 ▶).

## Supplementary Material

Crystal structure: contains datablock(s) global, I. DOI: 10.1107/S1600536812029194/hb6876sup1.cif

Structure factors: contains datablock(s) I. DOI: 10.1107/S1600536812029194/hb6876Isup2.hkl

Supplementary material file. DOI: 10.1107/S1600536812029194/hb6876Isup3.cml

Additional supplementary materials:  crystallographic information; 3D view; checkCIF report

## Figures and Tables

**Table 1:** Hydrogen-bond geometry (Å, °) *Cg*2, *Cg*3 and *Cg*4 are the centroids of the C8*A*–C13*A*, C15*A*–C20*A* and C8*B*–C13*B* rings, respectively.

*D*—H⋯*A*	*D*—H	H⋯*A*	*D*⋯*A*	*D*—H⋯*A*
C17*A*—H17*A*⋯O4*B* ^i^	0.95	2.49	3.2100 (16)	133
C9*A*—H9*AA*⋯*Cg*4^ii^	0.95	2.68	3.5487 (13)	152
C16*A*—H16*A*⋯*Cg*2^i^	0.95	2.68	3.5161 (13)	147
C20*B*—H20*B*⋯*Cg*3^iii^	0.95	2.87	3.7013 (14)	146

Symmetry codes: (i) 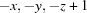; (ii) 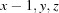; (iii) 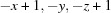.

**Fig. 1.**
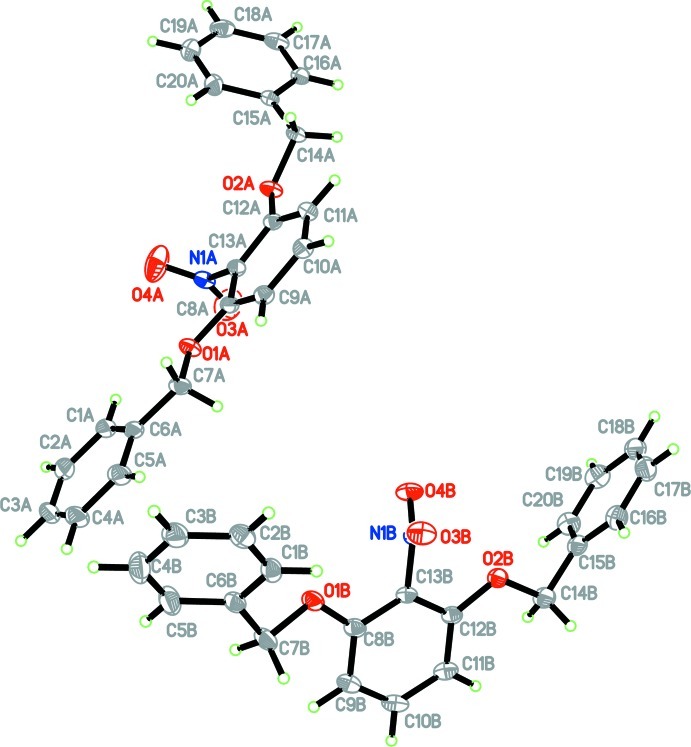
The molecular structure of the title compound, showing 50% probability displacement ellipsoids.

**Fig. 2.**
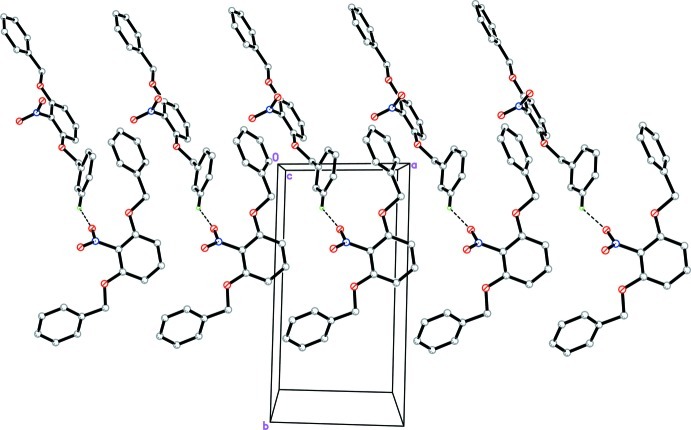
The crystal packing of the title compound, showing the formation of the inversion dimers. For the sake of clarity, those H atoms not involved in the intermolecular interactions (dashed lines) have been omitted.

## supplementary crystallographic information

### Comment

1-((3-(Benzyloxy)-2-nitrophenoxy)methyl)benzene derivatives are extensively used in Medicinal Chemistry as important intermediates for many pharmaceutical products (Altmann *et al.*, 2004). 3-(Benzyloxy)-2-nitrophenol is used as intermediate for the synthesis of anticancer products and many natural products as well (Ohkubo *et al.*, 1997). As part of our studies in this area, we hereby report the crystal structure of the title compound.

The asymmetric unit of the title compound (Fig. 1), consists of two crystallographically independent molecules, *A* and *B*. Bond lengths and angles are within normal ranges (Naveenkumar *et al.*, 2009; Fun *et al.*, 2011; Ren & Wang, 2012). In molecule *A*, the central benzene ring (C8A–C13A) forms dihedral angles of 2.26 (6) and 58.68 (6)°, respectively, with the terminal benzene rings (C1A–C6A & C15A–C20A). The dihedral angle between the terminal benzene rings is 56.45 (6)°. The corresponding values in molecule *B* are 35.17 (6), 70.97 (6) and 69.62 (6)°, respectively.

The crystal structure is shown in Fig. 2. The molecules are linked together with another neighbouring molecules *via* C17A—H17A···O4B hydrogen bonds (Table 1) to form inversion dimers. C—H···π interactions (Table 1) and π–π interactions of *Cg*1···*Cg*1 = 3.7113 (8) Å (symmetry code: 1 - *x*, 1 - *y*, 1 - *z*) and *Cg*1···*Cg*2 = 3.7216 (7) Å (symmetry code: -*x*, 1 - *y*, 1 - *z*) link the molecules into a three-dimensional network. [*Cg*1, *Cg*2, *Cg*3 and *Cg*4 are the centroids of the C1A–C6A, C8A–C13A, C15A–C20A and C8B–C13B rings, respectively].

### Experimental

To a stirred solution of 3-(benzyloxy)-2-nitrophenol (1 g, 0.006 mol) in acetonitrile (20 ml) was added potassium carbonate (0.89 g, 0.006 mol) benzyl bromide (1.1 g, 0.006 mol) drop-wise at 273 K. The reaction mixture was stirred at room temperature for 2 h. Mass analysis of crude reaction mixture confirms the completion of the reaction. The reaction mixture was concentrated and the residue was purified by column chromatography to get title compound, which was recrystallized using acetone to get orange plates. Yield: 55%, *M.p.* 351–353 K.

### Refinement

All the H atoms were positioned geometrically [C–H = 0.95 or 0.99 Å] and refined using a riding model with *U*_iso_(H) = 1.2 *U*_eq_(C). Three outliers were omitted (-1 1 0, -4 2 0 and 2 - 6 9) in the final refinement.

### Crystal data

**Table:**

C_20_H_17_NO_4_	*Z* = 4
*M**_r_* = 335.35	*F*(000) = 704
Triclinic, *P*1	*D*_x_ = 1.340 Mg m^−^^3^
Hall symbol: -P 1	Mo *K*α radiation, λ = 0.71073 Å
*a* = 7.6150 (4) Å	Cell parameters from 9997 reflections
*b* = 14.6248 (7) Å	θ = 2.7–30.1°
*c* = 15.2915 (8) Å	µ = 0.09 mm^−^^1^
α = 94.706 (1)°	*T* = 100 K
β = 101.627 (1)°	Plate, orange
γ = 90.572 (1)°	0.26 × 0.19 × 0.09 mm
*V* = 1661.80 (15) Å^3^	

### Data collection

**Table:**

Bruker SMART APEXII DUO CCD diffractometer	9716 independent reflections
Radiation source: fine-focus sealed tube	7508 reflections with *I* > 2σ(*I*)
Graphite monochromator	*R*_int_ = 0.032
φ and ω scans	θ_max_ = 30.1°, θ_min_ = 1.4°
Absorption correction: multi-scan (*SADABS*; Bruker, 2009)	*h* = −10→10
*T*_min_ = 0.976, *T*_max_ = 0.992	*k* = −20→20
35546 measured reflections	*l* = −21→21

### Refinement

**Table:**

Refinement on *F*^2^	Primary atom site location: structure-invariant direct methods
Least-squares matrix: full	Secondary atom site location: difference Fourier map
*R*[*F*^2^ > 2σ(*F*^2^)] = 0.042	Hydrogen site location: inferred from neighbouring sites
*wR*(*F*^2^) = 0.117	H-atom parameters constrained
*S* = 1.03	*w* = 1/[σ^2^(*F*_o_^2^) + (0.054*P*)^2^ + 0.4706*P*] where *P* = (*F*_o_^2^ + 2*F*_c_^2^)/3
9716 reflections	(Δ/σ)_max_ < 0.001
451 parameters	Δρ_max_ = 0.38 e Å^−^^3^
0 restraints	Δρ_min_ = −0.29 e Å^−^^3^

### Special details

**Table:**

Experimental. The crystal was placed in the cold stream of an Oxford Cryosystems Cobra open-flow nitrogen cryostat (Cosier & Glazer, 1986) operating at 100.0 (1) K.
Geometry. All e.s.d.'s (except the e.s.d. in the dihedral angle between two l.s. planes) are estimated using the full covariance matrix. The cell e.s.d.'s are taken into account individually in the estimation of e.s.d.'s in distances, angles and torsion angles; correlations between e.s.d.'s in cell parameters are only used when they are defined by crystal symmetry. An approximate (isotropic) treatment of cell e.s.d.'s is used for estimating e.s.d.'s involving l.s. planes.
Refinement. Refinement of *F*^2^ against ALL reflections. The weighted *R*-factor *wR* and goodness of fit *S* are based on *F*^2^, conventional *R*-factors *R* are based on *F*, with *F* set to zero for negative *F*^2^. The threshold expression of *F*^2^ > σ(*F*^2^) is used only for calculating *R*-factors(gt) *etc*. and is not relevant to the choice of reflections for refinement. *R*-factors based on *F*^2^ are statistically about twice as large as those based on *F*, and *R*- factors based on ALL data will be even larger.

### Fractional atomic coordinates and isotropic or equivalent isotropic displacement parameters (Å^2^)

**Table:**

	*x*	*y*	*z*	*U*_iso_*/*U*_eq_	
O1A	0.13559 (11)	0.36930 (5)	0.49220 (6)	0.02214 (17)	
O2A	−0.10558 (10)	0.09202 (5)	0.55126 (5)	0.02063 (17)	
O3A	0.25107 (14)	0.20447 (9)	0.62840 (7)	0.0468 (3)	
O4A	0.04238 (16)	0.28956 (9)	0.65945 (7)	0.0513 (3)	
N1A	0.10941 (13)	0.24213 (7)	0.60732 (7)	0.0215 (2)	
C1A	0.32496 (16)	0.52370 (8)	0.57710 (9)	0.0262 (2)	
H1AA	0.3024	0.4741	0.6101	0.031*	
C2A	0.42369 (17)	0.60071 (9)	0.62090 (11)	0.0336 (3)	
H2AA	0.4673	0.6039	0.6838	0.040*	
C3A	0.45841 (18)	0.67279 (9)	0.57274 (12)	0.0381 (4)	
H3AA	0.5258	0.7253	0.6028	0.046*	
C4A	0.39539 (19)	0.66844 (9)	0.48159 (12)	0.0374 (3)	
H4AA	0.4204	0.7176	0.4487	0.045*	
C5A	0.29527 (17)	0.59230 (8)	0.43760 (10)	0.0302 (3)	
H5AA	0.2509	0.5898	0.3747	0.036*	
C6A	0.25959 (15)	0.51938 (8)	0.48546 (9)	0.0230 (2)	
C7A	0.14950 (15)	0.43950 (8)	0.43354 (8)	0.0218 (2)	
H7AA	0.0283	0.4599	0.4070	0.026*	
H7AB	0.2068	0.4148	0.3842	0.026*	
C8A	0.03602 (14)	0.29318 (7)	0.45495 (8)	0.0185 (2)	
C9A	−0.04526 (15)	0.27616 (8)	0.36507 (8)	0.0201 (2)	
H9AA	−0.0334	0.3195	0.3235	0.024*	
C10A	−0.14422 (15)	0.19488 (8)	0.33674 (8)	0.0206 (2)	
H10A	−0.1983	0.1832	0.2751	0.025*	
C11A	−0.16659 (15)	0.13023 (8)	0.39534 (8)	0.0196 (2)	
H11A	−0.2345	0.0752	0.3741	0.024*	
C12A	−0.08803 (14)	0.14722 (7)	0.48569 (7)	0.0176 (2)	
C13A	0.01607 (14)	0.22707 (7)	0.51330 (7)	0.0176 (2)	
C14A	−0.24977 (15)	0.02361 (8)	0.52786 (8)	0.0198 (2)	
H14A	−0.2090	−0.0319	0.4969	0.024*	
H14B	−0.3530	0.0480	0.4870	0.024*	
C15A	−0.30454 (14)	−0.00015 (7)	0.61225 (8)	0.0179 (2)	
C16A	−0.29037 (15)	−0.08909 (8)	0.63851 (8)	0.0207 (2)	
H16A	−0.2380	−0.1348	0.6049	0.025*	
C17A	−0.35264 (16)	−0.11121 (9)	0.71380 (9)	0.0260 (3)	
H17A	−0.3434	−0.1721	0.7313	0.031*	
C18A	−0.42811 (17)	−0.04469 (10)	0.76329 (9)	0.0297 (3)	
H18A	−0.4709	−0.0600	0.8146	0.036*	
C19A	−0.44114 (19)	0.04415 (10)	0.73792 (9)	0.0322 (3)	
H19A	−0.4920	0.0900	0.7722	0.039*	
C20A	−0.38016 (17)	0.06621 (8)	0.66276 (9)	0.0257 (2)	
H20A	−0.3900	0.1272	0.6455	0.031*	
O1B	0.61240 (11)	0.48372 (5)	0.17237 (6)	0.02478 (18)	
O2B	0.81078 (10)	0.19193 (5)	0.10404 (6)	0.02094 (17)	
O3B	0.44330 (11)	0.33094 (6)	0.04889 (6)	0.0286 (2)	
O4B	0.49167 (11)	0.25710 (6)	0.16793 (6)	0.02714 (19)	
N1B	0.54058 (12)	0.30732 (6)	0.11650 (7)	0.01836 (18)	
C1B	0.31381 (17)	0.57666 (8)	0.08217 (8)	0.0234 (2)	
H1BA	0.3444	0.5245	0.0477	0.028*	
C2B	0.14345 (17)	0.61162 (8)	0.06114 (8)	0.0257 (2)	
H2BA	0.0577	0.5829	0.0126	0.031*	
C3B	0.09729 (18)	0.68790 (9)	0.11025 (10)	0.0316 (3)	
H3BA	−0.0192	0.7121	0.0951	0.038*	
C4B	0.2218 (2)	0.72864 (10)	0.18152 (11)	0.0401 (4)	
H4BA	0.1905	0.7809	0.2157	0.048*	
C5B	0.39273 (19)	0.69359 (9)	0.20353 (10)	0.0342 (3)	
H5BA	0.4774	0.7217	0.2529	0.041*	
C6B	0.44001 (16)	0.61761 (8)	0.15347 (8)	0.0223 (2)	
C7B	0.62756 (17)	0.58239 (8)	0.17359 (9)	0.0262 (2)	
H7BA	0.6945	0.5995	0.1279	0.031*	
H7BB	0.6923	0.6090	0.2331	0.031*	
C8B	0.76005 (15)	0.43423 (8)	0.16589 (8)	0.0209 (2)	
C9B	0.93703 (16)	0.46798 (8)	0.18596 (8)	0.0243 (2)	
H9BA	0.9639	0.5306	0.2065	0.029*	
C10B	1.07298 (16)	0.40807 (8)	0.17527 (8)	0.0249 (2)	
H10B	1.1932	0.4313	0.1877	0.030*	
C11B	1.04041 (15)	0.31547 (8)	0.14720 (8)	0.0224 (2)	
H11B	1.1365	0.2765	0.1401	0.027*	
C12B	0.86381 (14)	0.28059 (7)	0.12962 (7)	0.0186 (2)	
C13B	0.72783 (14)	0.34141 (7)	0.13854 (7)	0.0180 (2)	
C14B	0.94773 (16)	0.12652 (8)	0.09238 (10)	0.0267 (3)	
H14C	1.0539	0.1373	0.1417	0.032*	
H14D	0.9856	0.1330	0.0349	0.032*	
C15B	0.86818 (15)	0.03228 (8)	0.09284 (8)	0.0223 (2)	
C16B	0.74639 (17)	−0.00707 (9)	0.01872 (9)	0.0271 (3)	
H16B	0.7152	0.0253	−0.0334	0.033*	
C17B	0.67035 (18)	−0.09329 (9)	0.02052 (10)	0.0307 (3)	
H17B	0.5868	−0.1196	−0.0302	0.037*	
C18B	0.71567 (17)	−0.14099 (9)	0.09577 (10)	0.0293 (3)	
H18B	0.6627	−0.1999	0.0969	0.035*	
C19B	0.83853 (18)	−0.10289 (9)	0.16962 (10)	0.0313 (3)	
H19B	0.8711	−0.1360	0.2211	0.038*	
C20B	0.91417 (17)	−0.01623 (9)	0.16831 (9)	0.0281 (3)	
H20B	0.9976	0.0100	0.2192	0.034*	

### Atomic displacement parameters (Å^2^)

**Table:**

	*U*^11^	*U*^22^	*U*^33^	*U*^12^	*U*^13^	*U*^23^
O1A	0.0237 (4)	0.0180 (4)	0.0246 (4)	−0.0058 (3)	0.0041 (3)	0.0039 (3)
O2A	0.0195 (4)	0.0217 (4)	0.0198 (4)	−0.0067 (3)	0.0004 (3)	0.0063 (3)
O3A	0.0319 (5)	0.0672 (8)	0.0343 (6)	0.0153 (5)	−0.0084 (4)	−0.0002 (5)
O4A	0.0507 (7)	0.0733 (8)	0.0250 (5)	0.0227 (6)	0.0016 (5)	−0.0121 (5)
N1A	0.0211 (4)	0.0216 (5)	0.0209 (5)	−0.0061 (4)	0.0016 (4)	0.0036 (4)
C1A	0.0207 (5)	0.0216 (5)	0.0378 (7)	−0.0006 (4)	0.0106 (5)	−0.0007 (5)
C2A	0.0237 (6)	0.0299 (6)	0.0467 (8)	−0.0024 (5)	0.0119 (6)	−0.0109 (6)
C3A	0.0257 (6)	0.0200 (6)	0.0697 (11)	−0.0039 (5)	0.0176 (7)	−0.0096 (6)
C4A	0.0325 (7)	0.0162 (5)	0.0684 (11)	0.0007 (5)	0.0208 (7)	0.0072 (6)
C5A	0.0267 (6)	0.0190 (5)	0.0483 (8)	0.0026 (5)	0.0132 (6)	0.0085 (5)
C6A	0.0170 (5)	0.0161 (5)	0.0382 (7)	0.0015 (4)	0.0109 (5)	0.0028 (4)
C7A	0.0193 (5)	0.0189 (5)	0.0287 (6)	−0.0008 (4)	0.0065 (4)	0.0072 (4)
C8A	0.0157 (5)	0.0167 (5)	0.0239 (6)	−0.0002 (4)	0.0053 (4)	0.0021 (4)
C9A	0.0206 (5)	0.0196 (5)	0.0213 (5)	0.0012 (4)	0.0061 (4)	0.0056 (4)
C10A	0.0208 (5)	0.0229 (5)	0.0182 (5)	0.0017 (4)	0.0035 (4)	0.0033 (4)
C11A	0.0193 (5)	0.0185 (5)	0.0204 (5)	−0.0015 (4)	0.0024 (4)	0.0019 (4)
C12A	0.0163 (5)	0.0174 (5)	0.0199 (5)	0.0005 (4)	0.0044 (4)	0.0044 (4)
C13A	0.0159 (4)	0.0188 (5)	0.0178 (5)	−0.0004 (4)	0.0025 (4)	0.0023 (4)
C14A	0.0186 (5)	0.0180 (5)	0.0223 (6)	−0.0049 (4)	0.0030 (4)	0.0025 (4)
C15A	0.0155 (4)	0.0173 (5)	0.0203 (5)	−0.0031 (4)	0.0021 (4)	0.0018 (4)
C16A	0.0180 (5)	0.0184 (5)	0.0251 (6)	−0.0013 (4)	0.0027 (4)	0.0025 (4)
C17A	0.0229 (5)	0.0266 (6)	0.0273 (6)	−0.0076 (4)	−0.0003 (5)	0.0097 (5)
C18A	0.0273 (6)	0.0413 (7)	0.0209 (6)	−0.0120 (5)	0.0058 (5)	0.0036 (5)
C19A	0.0345 (7)	0.0337 (7)	0.0304 (7)	−0.0027 (5)	0.0145 (5)	−0.0059 (5)
C20A	0.0293 (6)	0.0188 (5)	0.0298 (6)	0.0011 (4)	0.0082 (5)	0.0008 (4)
O1B	0.0241 (4)	0.0144 (4)	0.0377 (5)	0.0001 (3)	0.0098 (4)	0.0045 (3)
O2B	0.0170 (4)	0.0180 (4)	0.0286 (4)	0.0028 (3)	0.0059 (3)	0.0027 (3)
O3B	0.0220 (4)	0.0330 (5)	0.0293 (5)	−0.0011 (3)	−0.0010 (3)	0.0101 (4)
O4B	0.0240 (4)	0.0280 (4)	0.0327 (5)	−0.0033 (3)	0.0100 (4)	0.0121 (4)
N1B	0.0172 (4)	0.0153 (4)	0.0236 (5)	0.0010 (3)	0.0062 (3)	0.0026 (3)
C1B	0.0317 (6)	0.0186 (5)	0.0208 (6)	0.0006 (4)	0.0069 (5)	0.0024 (4)
C2B	0.0302 (6)	0.0223 (5)	0.0242 (6)	−0.0025 (5)	0.0032 (5)	0.0063 (4)
C3B	0.0268 (6)	0.0237 (6)	0.0455 (8)	0.0018 (5)	0.0089 (6)	0.0051 (5)
C4B	0.0364 (7)	0.0284 (7)	0.0531 (9)	0.0049 (6)	0.0095 (7)	−0.0129 (6)
C5B	0.0332 (7)	0.0261 (6)	0.0390 (8)	−0.0007 (5)	0.0024 (6)	−0.0099 (5)
C6B	0.0272 (6)	0.0154 (5)	0.0252 (6)	−0.0016 (4)	0.0070 (5)	0.0038 (4)
C7B	0.0276 (6)	0.0145 (5)	0.0357 (7)	−0.0020 (4)	0.0043 (5)	0.0027 (4)
C8B	0.0221 (5)	0.0187 (5)	0.0231 (6)	−0.0005 (4)	0.0054 (4)	0.0063 (4)
C9B	0.0244 (5)	0.0203 (5)	0.0280 (6)	−0.0048 (4)	0.0033 (5)	0.0065 (4)
C10B	0.0194 (5)	0.0277 (6)	0.0276 (6)	−0.0053 (4)	0.0018 (4)	0.0112 (5)
C11B	0.0174 (5)	0.0263 (6)	0.0249 (6)	0.0008 (4)	0.0046 (4)	0.0100 (4)
C12B	0.0187 (5)	0.0193 (5)	0.0190 (5)	0.0003 (4)	0.0047 (4)	0.0059 (4)
C13B	0.0162 (5)	0.0184 (5)	0.0204 (5)	−0.0018 (4)	0.0044 (4)	0.0058 (4)
C14B	0.0192 (5)	0.0222 (6)	0.0406 (7)	0.0054 (4)	0.0100 (5)	0.0032 (5)
C15B	0.0185 (5)	0.0201 (5)	0.0298 (6)	0.0059 (4)	0.0081 (4)	0.0021 (4)
C16B	0.0288 (6)	0.0263 (6)	0.0265 (6)	0.0057 (5)	0.0054 (5)	0.0033 (5)
C17B	0.0301 (6)	0.0269 (6)	0.0334 (7)	0.0011 (5)	0.0051 (5)	−0.0040 (5)
C18B	0.0290 (6)	0.0212 (6)	0.0405 (8)	0.0032 (5)	0.0140 (5)	0.0015 (5)
C19B	0.0330 (7)	0.0300 (6)	0.0337 (7)	0.0066 (5)	0.0095 (5)	0.0115 (5)
C20B	0.0245 (6)	0.0295 (6)	0.0293 (7)	0.0037 (5)	0.0027 (5)	0.0039 (5)

### Geometric parameters (Å, º)

**Table:**

O1A—C8A	1.3579 (13)	O1B—C8B	1.3591 (14)
O1A—C7A	1.4343 (13)	O1B—C7B	1.4448 (13)
O2A—C12A	1.3636 (13)	O2B—C12B	1.3565 (13)
O2A—C14A	1.4476 (13)	O2B—C14B	1.4495 (13)
O3A—N1A	1.2131 (14)	O3B—N1B	1.2214 (12)
O4A—N1A	1.2073 (14)	O4B—N1B	1.2263 (12)
N1A—C13A	1.4675 (14)	N1B—C13B	1.4697 (14)
C1A—C6A	1.3854 (19)	C1B—C2B	1.3856 (17)
C1A—C2A	1.3924 (18)	C1B—C6B	1.3892 (17)
C1A—H1AA	0.9500	C1B—H1BA	0.9500
C2A—C3A	1.387 (2)	C2B—C3B	1.3815 (18)
C2A—H2AA	0.9500	C2B—H2BA	0.9500
C3A—C4A	1.376 (2)	C3B—C4B	1.381 (2)
C3A—H3AA	0.9500	C3B—H3BA	0.9500
C4A—C5A	1.388 (2)	C4B—C5B	1.391 (2)
C4A—H4AA	0.9500	C4B—H4BA	0.9500
C5A—C6A	1.3961 (17)	C5B—C6B	1.3892 (17)
C5A—H5AA	0.9500	C5B—H5BA	0.9500
C6A—C7A	1.5026 (16)	C6B—C7B	1.5045 (17)
C7A—H7AA	0.9900	C7B—H7BA	0.9900
C7A—H7AB	0.9900	C7B—H7BB	0.9900
C8A—C9A	1.3899 (16)	C8B—C13B	1.3916 (15)
C8A—C13A	1.3954 (15)	C8B—C9B	1.3962 (16)
C9A—C10A	1.3909 (16)	C9B—C10B	1.3889 (17)
C9A—H9AA	0.9500	C9B—H9BA	0.9500
C10A—C11A	1.3868 (15)	C10B—C11B	1.3909 (17)
C10A—H10A	0.9500	C10B—H10B	0.9500
C11A—C12A	1.3914 (16)	C11B—C12B	1.3997 (15)
C11A—H11A	0.9500	C11B—H11B	0.9500
C12A—C13A	1.3915 (15)	C12B—C13B	1.3919 (15)
C14A—C15A	1.4992 (16)	C14B—C15B	1.5010 (17)
C14A—H14A	0.9900	C14B—H14C	0.9900
C14A—H14B	0.9900	C14B—H14D	0.9900
C15A—C16A	1.3914 (15)	C15B—C20B	1.3907 (17)
C15A—C20A	1.3919 (16)	C15B—C16B	1.3910 (18)
C16A—C17A	1.3903 (17)	C16B—C17B	1.3869 (18)
C16A—H16A	0.9500	C16B—H16B	0.9500
C17A—C18A	1.3838 (19)	C17B—C18B	1.381 (2)
C17A—H17A	0.9500	C17B—H17B	0.9500
C18A—C19A	1.385 (2)	C18B—C19B	1.386 (2)
C18A—H18A	0.9500	C18B—H18B	0.9500
C19A—C20A	1.3833 (19)	C19B—C20B	1.3903 (19)
C19A—H19A	0.9500	C19B—H19B	0.9500
C20A—H20A	0.9500	C20B—H20B	0.9500
			
C8A—O1A—C7A	116.28 (9)	C8B—O1B—C7B	117.97 (9)
C12A—O2A—C14A	115.70 (8)	C12B—O2B—C14B	117.79 (9)
O4A—N1A—O3A	123.50 (11)	O3B—N1B—O4B	124.10 (10)
O4A—N1A—C13A	119.39 (10)	O3B—N1B—C13B	118.39 (9)
O3A—N1A—C13A	117.10 (10)	O4B—N1B—C13B	117.50 (9)
C6A—C1A—C2A	120.03 (12)	C2B—C1B—C6B	120.28 (11)
C6A—C1A—H1AA	120.0	C2B—C1B—H1BA	119.9
C2A—C1A—H1AA	120.0	C6B—C1B—H1BA	119.9
C3A—C2A—C1A	120.08 (14)	C3B—C2B—C1B	120.54 (12)
C3A—C2A—H2AA	120.0	C3B—C2B—H2BA	119.7
C1A—C2A—H2AA	120.0	C1B—C2B—H2BA	119.7
C4A—C3A—C2A	120.12 (13)	C4B—C3B—C2B	119.47 (12)
C4A—C3A—H3AA	119.9	C4B—C3B—H3BA	120.3
C2A—C3A—H3AA	119.9	C2B—C3B—H3BA	120.3
C3A—C4A—C5A	120.13 (13)	C3B—C4B—C5B	120.38 (13)
C3A—C4A—H4AA	119.9	C3B—C4B—H4BA	119.8
C5A—C4A—H4AA	119.9	C5B—C4B—H4BA	119.8
C4A—C5A—C6A	120.20 (14)	C6B—C5B—C4B	120.19 (13)
C4A—C5A—H5AA	119.9	C6B—C5B—H5BA	119.9
C6A—C5A—H5AA	119.9	C4B—C5B—H5BA	119.9
C1A—C6A—C5A	119.44 (12)	C5B—C6B—C1B	119.13 (11)
C1A—C6A—C7A	123.28 (11)	C5B—C6B—C7B	120.56 (11)
C5A—C6A—C7A	117.28 (12)	C1B—C6B—C7B	120.26 (11)
O1A—C7A—C6A	109.51 (10)	O1B—C7B—C6B	107.15 (9)
O1A—C7A—H7AA	109.8	O1B—C7B—H7BA	110.3
C6A—C7A—H7AA	109.8	C6B—C7B—H7BA	110.3
O1A—C7A—H7AB	109.8	O1B—C7B—H7BB	110.3
C6A—C7A—H7AB	109.8	C6B—C7B—H7BB	110.3
H7AA—C7A—H7AB	108.2	H7BA—C7B—H7BB	108.5
O1A—C8A—C9A	125.74 (10)	O1B—C8B—C13B	115.54 (10)
O1A—C8A—C13A	115.96 (10)	O1B—C8B—C9B	125.85 (11)
C9A—C8A—C13A	118.30 (10)	C13B—C8B—C9B	118.59 (10)
C8A—C9A—C10A	119.19 (10)	C10B—C9B—C8B	118.60 (11)
C8A—C9A—H9AA	120.4	C10B—C9B—H9BA	120.7
C10A—C9A—H9AA	120.4	C8B—C9B—H9BA	120.7
C11A—C10A—C9A	122.27 (11)	C9B—C10B—C11B	122.72 (11)
C11A—C10A—H10A	118.9	C9B—C10B—H10B	118.6
C9A—C10A—H10A	118.9	C11B—C10B—H10B	118.6
C10A—C11A—C12A	119.00 (10)	C10B—C11B—C12B	118.96 (11)
C10A—C11A—H11A	120.5	C10B—C11B—H11B	120.5
C12A—C11A—H11A	120.5	C12B—C11B—H11B	120.5
O2A—C12A—C11A	125.33 (10)	O2B—C12B—C13B	115.94 (9)
O2A—C12A—C13A	116.09 (10)	O2B—C12B—C11B	126.05 (10)
C11A—C12A—C13A	118.58 (10)	C13B—C12B—C11B	118.01 (10)
C12A—C13A—C8A	122.56 (10)	C8B—C13B—C12B	123.08 (10)
C12A—C13A—N1A	118.74 (9)	C8B—C13B—N1B	117.85 (9)
C8A—C13A—N1A	118.70 (9)	C12B—C13B—N1B	119.06 (10)
O2A—C14A—C15A	108.45 (9)	O2B—C14B—C15B	107.41 (9)
O2A—C14A—H14A	110.0	O2B—C14B—H14C	110.2
C15A—C14A—H14A	110.0	C15B—C14B—H14C	110.2
O2A—C14A—H14B	110.0	O2B—C14B—H14D	110.2
C15A—C14A—H14B	110.0	C15B—C14B—H14D	110.2
H14A—C14A—H14B	108.4	H14C—C14B—H14D	108.5
C16A—C15A—C20A	119.12 (11)	C20B—C15B—C16B	119.24 (11)
C16A—C15A—C14A	121.02 (10)	C20B—C15B—C14B	120.06 (11)
C20A—C15A—C14A	119.76 (10)	C16B—C15B—C14B	120.69 (11)
C17A—C16A—C15A	120.20 (11)	C17B—C16B—C15B	120.30 (12)
C17A—C16A—H16A	119.9	C17B—C16B—H16B	119.8
C15A—C16A—H16A	119.9	C15B—C16B—H16B	119.8
C18A—C17A—C16A	120.13 (11)	C18B—C17B—C16B	120.26 (13)
C18A—C17A—H17A	119.9	C18B—C17B—H17B	119.9
C16A—C17A—H17A	119.9	C16B—C17B—H17B	119.9
C17A—C18A—C19A	119.93 (12)	C17B—C18B—C19B	119.92 (12)
C17A—C18A—H18A	120.0	C17B—C18B—H18B	120.0
C19A—C18A—H18A	120.0	C19B—C18B—H18B	120.0
C20A—C19A—C18A	120.04 (12)	C18B—C19B—C20B	120.00 (12)
C20A—C19A—H19A	120.0	C18B—C19B—H19B	120.0
C18A—C19A—H19A	120.0	C20B—C19B—H19B	120.0
C19A—C20A—C15A	120.57 (11)	C19B—C20B—C15B	120.27 (12)
C19A—C20A—H20A	119.7	C19B—C20B—H20B	119.9
C15A—C20A—H20A	119.7	C15B—C20B—H20B	119.9
			
C6A—C1A—C2A—C3A	−0.70 (18)	C6B—C1B—C2B—C3B	0.48 (18)
C1A—C2A—C3A—C4A	0.03 (19)	C1B—C2B—C3B—C4B	−0.8 (2)
C2A—C3A—C4A—C5A	0.6 (2)	C2B—C3B—C4B—C5B	0.4 (2)
C3A—C4A—C5A—C6A	−0.65 (19)	C3B—C4B—C5B—C6B	0.5 (2)
C2A—C1A—C6A—C5A	0.69 (17)	C4B—C5B—C6B—C1B	−0.8 (2)
C2A—C1A—C6A—C7A	−178.96 (11)	C4B—C5B—C6B—C7B	176.62 (13)
C4A—C5A—C6A—C1A	−0.02 (18)	C2B—C1B—C6B—C5B	0.36 (18)
C4A—C5A—C6A—C7A	179.65 (11)	C2B—C1B—C6B—C7B	−177.11 (11)
C8A—O1A—C7A—C6A	178.60 (9)	C8B—O1B—C7B—C6B	163.48 (10)
C1A—C6A—C7A—O1A	−3.76 (15)	C5B—C6B—C7B—O1B	135.13 (12)
C5A—C6A—C7A—O1A	176.59 (9)	C1B—C6B—C7B—O1B	−47.45 (15)
C7A—O1A—C8A—C9A	3.17 (15)	C7B—O1B—C8B—C13B	−161.68 (10)
C7A—O1A—C8A—C13A	−177.09 (9)	C7B—O1B—C8B—C9B	19.83 (18)
O1A—C8A—C9A—C10A	−179.70 (10)	O1B—C8B—C9B—C10B	−179.71 (11)
C13A—C8A—C9A—C10A	0.56 (16)	C13B—C8B—C9B—C10B	1.85 (17)
C8A—C9A—C10A—C11A	0.73 (17)	C8B—C9B—C10B—C11B	−1.37 (19)
C9A—C10A—C11A—C12A	0.23 (17)	C9B—C10B—C11B—C12B	−0.57 (18)
C14A—O2A—C12A—C11A	−15.49 (15)	C14B—O2B—C12B—C13B	178.94 (10)
C14A—O2A—C12A—C13A	163.81 (9)	C14B—O2B—C12B—C11B	−0.86 (16)
C10A—C11A—C12A—O2A	176.84 (10)	C10B—C11B—C12B—O2B	−178.27 (11)
C10A—C11A—C12A—C13A	−2.44 (16)	C10B—C11B—C12B—C13B	1.93 (17)
O2A—C12A—C13A—C8A	−175.50 (9)	O1B—C8B—C13B—C12B	−179.07 (10)
C11A—C12A—C13A—C8A	3.85 (16)	C9B—C8B—C13B—C12B	−0.46 (17)
O2A—C12A—C13A—N1A	4.89 (14)	O1B—C8B—C13B—N1B	2.22 (15)
C11A—C12A—C13A—N1A	−175.76 (10)	C9B—C8B—C13B—N1B	−179.17 (10)
O1A—C8A—C13A—C12A	177.34 (10)	O2B—C12B—C13B—C8B	178.73 (10)
C9A—C8A—C13A—C12A	−2.89 (16)	C11B—C12B—C13B—C8B	−1.46 (17)
O1A—C8A—C13A—N1A	−3.04 (14)	O2B—C12B—C13B—N1B	−2.58 (15)
C9A—C8A—C13A—N1A	176.72 (10)	C11B—C12B—C13B—N1B	177.24 (10)
O4A—N1A—C13A—C12A	−97.23 (14)	O3B—N1B—C13B—C8B	69.71 (14)
O3A—N1A—C13A—C12A	81.92 (14)	O4B—N1B—C13B—C8B	−109.84 (12)
O4A—N1A—C13A—C8A	83.14 (14)	O3B—N1B—C13B—C12B	−109.06 (12)
O3A—N1A—C13A—C8A	−97.71 (13)	O4B—N1B—C13B—C12B	71.39 (14)
C12A—O2A—C14A—C15A	−154.61 (9)	C12B—O2B—C14B—C15B	162.67 (10)
O2A—C14A—C15A—C16A	−118.80 (11)	O2B—C14B—C15B—C20B	−103.79 (12)
O2A—C14A—C15A—C20A	64.84 (13)	O2B—C14B—C15B—C16B	75.37 (14)
C20A—C15A—C16A—C17A	0.58 (17)	C20B—C15B—C16B—C17B	0.69 (18)
C14A—C15A—C16A—C17A	−175.80 (10)	C14B—C15B—C16B—C17B	−178.48 (11)
C15A—C16A—C17A—C18A	−0.37 (17)	C15B—C16B—C17B—C18B	−0.35 (19)
C16A—C17A—C18A—C19A	−0.19 (19)	C16B—C17B—C18B—C19B	−0.43 (19)
C17A—C18A—C19A—C20A	0.5 (2)	C17B—C18B—C19B—C20B	0.85 (19)
C18A—C19A—C20A—C15A	−0.3 (2)	C18B—C19B—C20B—C15B	−0.50 (19)
C16A—C15A—C20A—C19A	−0.23 (18)	C16B—C15B—C20B—C19B	−0.27 (18)
C14A—C15A—C20A—C19A	176.19 (11)	C14B—C15B—C20B—C19B	178.91 (11)

### Hydrogen-bond geometry (Å, º)

*Cg*2 , *Cg*3 and *Cg*4 are the centroids of the C8A–C13A, C15A–C20A and C8B–C13B rings, respectively.

**Table:**

*D*—H···*A*	*D*—H	H···*A*	*D*···*A*	*D*—H···*A*
C17*A*—H17*A*···O4*B*^i^	0.95	2.49	3.2100 (16)	133
C9*A*—H9*AA*···*Cg*4^ii^	0.95	2.68	3.5487 (13)	152
C16*A*—H16*A*···*Cg*2^i^	0.95	2.68	3.5161 (13)	147
C20*B*—H20*B*···*Cg*3^iii^	0.95	2.87	3.7013 (14)	146

Symmetry codes: (i) −*x*, −*y*, −*z*+1; (ii) *x*−1, *y*, *z*; (iii) −*x*+1, −*y*, −*z*+1.
